# Optimized ^68^Ga-Labeled Urea-Based PSMA-Targeted PET Tracers for Prostate Cancer

**DOI:** 10.3390/ph15081001

**Published:** 2022-08-14

**Authors:** Yitian Wu, Xiaojun Zhang, Ying Zhang, Baixuan Xu, Jiahe Tian, Jinming Zhang

**Affiliations:** 1 Department of Nuclear Medicine, Chinese PLA General Hospital, Beijing 100853, China; 2 Department of Oncology, The First Medical Center, Chinese PLA General Hospital, Beijing 100853, China

**Keywords:** PSMA, prostate cancer, PET imaging, ^68^Ga, theranostic

## Abstract

Prostate-specific membrane antigen (PSMA)-targeting radiopharmaceuticals have become some of the most promising tools for the diagnosis and therapy prostate cancer (PCa). The structure of existing PSMA-targeted PET tracers still needs to be optimized to improve their pharmacokinetic properties and tumor-to-background ratio. In this study, we modified the structure of a well-studied PSMA tracer, and six novel tracers with variable hydrophilicity and pharmacokinetics were developed and evaluated both in vitro and in vivo. All of the novel tracers showed high hydrophilicity (log *p* = −2.99 ± 0.33 to −3.49 ± 0.01), rapid clearance rates (elimination half-times = 15.55 to 35.97 min), and high affinity for PSMA (Ki = 8.11 ± 0.49 to 42.40 ± 2.11 nM) in vitro. Specific cell binding and micro-PET experiments showed that [^68^Ga]Ga-PSMA-Q displayed the highest specific PSMA+ cell uptake (3.75 ± 0.35 IA%/10^6^ at 60 min), tumor uptake (SUVmax = 0.97 ± 0.24 at 60 min p.i.), and tumor-to-muscle ratio (59.33 ± 5.72 at 60 min p.i.), while the tumor-to-muscle ratio was much higher than that of [^68^Ga]Ga-PSMA-617. The results of this study validate the clinical potential of [^68^Ga]Ga-PSMA-Q for PET imaging and further targeted therapy of prostate cancer.

## 1. Introduction

Prostate cancer is the leading cause of death in elderly men, and millions of patients are newly diagnosed with prostate cancer (PCa) annually—especially in high-income countries and regions [1]. With economic development and the aging of the population, the incidence rate of PCa is increasing rapidly in China and other developing countries [2]. The treatment options and prognosis for PCa patients are mainly related to the stage of progression [3,4]. Radical resection and radiotherapy with or without anti-hormonal treatment have been used in early localized prostate cancer as a curative therapy [5]. Treatment options and their efficacy for advanced or biochemical relapse (BCR) cases are limited [6,7,8,9,10]. Thus, accurate early diagnosis plays a crucial role for PCa patients. Prostate-specific membrane antigen (PSMA)-ligand PET/CT has achieved rapid development in the past few decades. Several PSMA PET tracers have been developed and used in clinical PCa diagnosis, such as [^68^Ga]Ga-PSMA-11 [11,12] and ^18^F-DCFPyL [13,14], which have already been approved by the FDA. The existing PSMA PET tracers showed a considerably high detection rate of PCa lesions. Although the inherent shortcomings of radionuclide ^68^Ga limit its use to some extent, the convenience of its radiolabeling and the similarity of the chemical coordination properties of ^68^Ga and ^177^Lu/^225^Ac have imbued ^68^Ga-labeled tracers with great research and clinical value [15,16,17,18]. As the most studied PSMA tracer, [^68^Ga]Ga-PSMA-11 exhibits a rapid clearance rate, leading to a satisfying tumor-to-background ratio. However, the ligand PSMA-11 cannot be radiolabeled with ^177^Lu for therapy, due to its structural constraints. PSMA-617, another agent with a very high affinity for PSMA, can be radiolabeled with ^68^Ga and ^177^Lu/^225^Ac, showing potential theranostic value for PCa [19,20,21]. [^177^Lu]Lu-PSMA-617 was approved by the U.S. Food and Drug Administration (FDA) on 23 March 2022.

However, the pharmacokinetic characteristics of PSMA-617 still need to be optimized in order to provide a better T/NT ratio in a shorter period and reduce the toxicity to healthy organs. The EANM standardized reporting guidelines still recommend [^68^Ga]Ga-PSMA-11 rather than [^68^Ga]Ga-PSMA-617 for the patient selection of [^177^Lu]Lu-PSMA-617 therapy and the evaluation of the therapy’s efficacy, due to the relatively slow clearance of [^68^Ga]Ga-PSMA-617 [22,23].

A previous study showed that the clearance rate of the compound can be accelerated by modifying the linker group. The faster the clearance rate of the radiopharmaceuticals, the better the therapeutic efficacy [19]. The improved clearance rate may bring us closer to the theranostics of [^68^Ga]Ga/[^177^Lu]Lu-PSMA-X on the premise of achieving tumor uptake comparable to that of [^68^Ga]Ga/[^177^Lu]Lu-PSMA-617, which was the ultimate goal of our study.

Here, we adopted two steps to modify the structure of PSMA-617: first, replace the naphthyl in PSMA-617 with different groups, developing three novel PSMA tracers: PSMA-Q (which contains a 3-quinoline with higher hydrophilicity than naphthyl), PSMA-4PY (which contains a 4-pyridyl with substantially high hydrophilicity), and PSMA-P (which contains a pyrene with lipophilicity); next, replace the linker group (cyclohexyl) of the selected compound (PSMA-Q) in the first step with propyl, butyl, and phenyl, respectively, developing another three novel ^68^Ga-labeled PSMA tracers: PSMA-Q-1 (which contains a propyl), PSMA-Q-2 (which contains a butyl), and PSMA-Q-3 (which contains a phenyl). All novel tracers were labeled with ^68^Ga for further evaluation, and the best tracer with favorable PSMA affinity and pharmacokinetic characteristics was selected through both in vitro and in vivo preclinical experiments.

## 2. Materials and Methods

### 2.1. General

All chemicals, reagents, and solvents for the synthesis and analysis were of analytical grade. All animal studies were performed according to the protocol approved by the Animal Care and Use Committee of the Chinese PLA General Hospital (approval number: S2020-127-01).

### 2.2. Chemical Synthesis, Radiolabeling, and Quality Control

**Figure 1:**
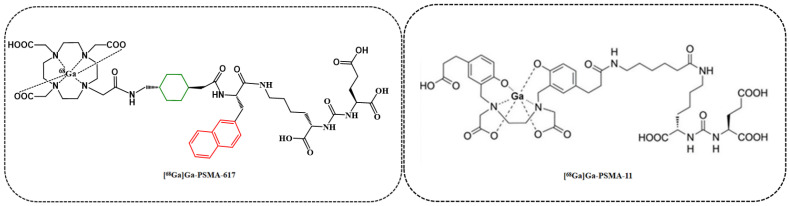

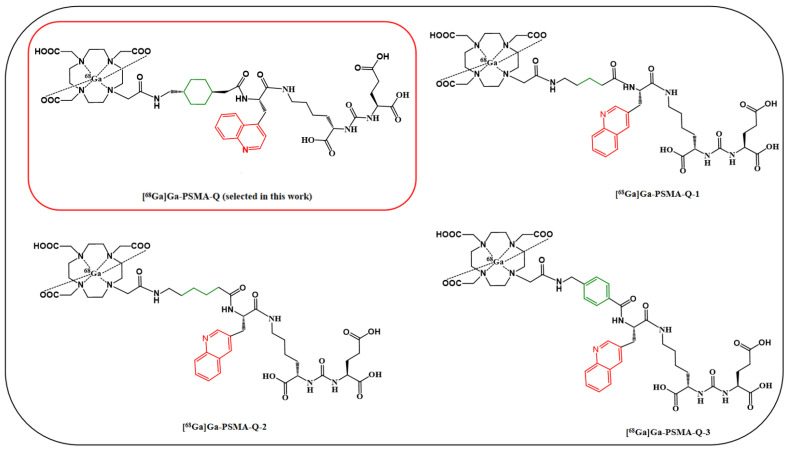
The chemical structures of [^68^Ga]Ga-PSMA-617, [^68^Ga]Ga-PSMA-11, [^68^Ga]Ga-PSMA-Q, [^68^Ga]Ga-PSMA-Q-1, [^68^Ga]Ga-PSMA-Q-2, and [^68^Ga]Ga-PSMA-Q-3.

**Scheme 1:**
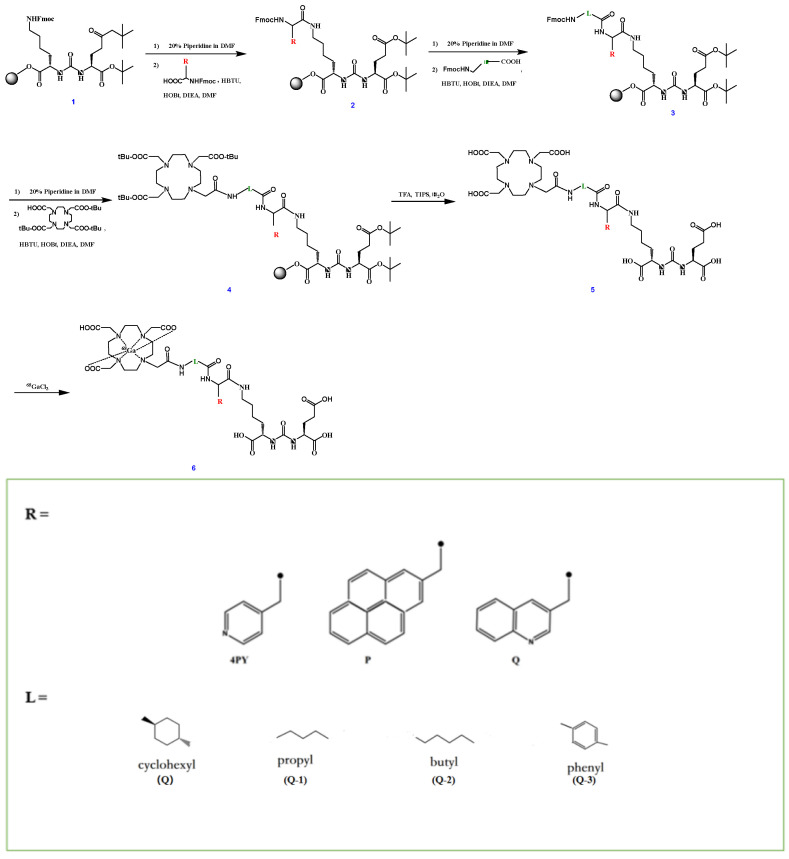
Synthesis of novel PSMA tracers.

The radiosynthesis routes of the novel tracers are shown in Scheme 1. The structures of PSMA-11, PSMA-617 and novel tracers are shown in Figure 1. The details of radiosynthesis and quality control are described in the Supplementary Materials.

### 2.3. In Vitro Studies

The partition coefficient of each novel radiotracer was determined using an octanol–water system (*v*/*v* = 1:1). The in vitro stability was determined using two systems (PBS and 5% BSA). The Ki value was measured by the NAALADase assay. The 22Rv1 and PC-3 cell lines were used for the cell-binding study and establishment of the tumor-bearing model (detailed information is shown in the Supplementary Materials).

### 2.4. Radiotoxicity, Pharmacokinetics, Biodistribution, and Micro-PET Imaging

The animal studies were approved by the institutional animal care and use committee of the Chinese PLA General Hospital. Healthy male ICR mice were injected with either a novel radiotracer (37 MBq in 150 μL) or saline (150 μL) for the radiotoxicity evaluation. Injection of a novel tracer, or of [^68^Ga]Ga-PSMA-617 or [^68^Ga]Ga-PSMA-11 (7.4 MBq in 150 μL), was administered intravenously via the tail into healthy male ICR mice for the pharmacokinetics studies. Biodistribution and micro-PET studies were performed on 22Rv1 and PC-3 xenograft models, and 2-PMPA was used as the PSMA blocker (all details are shown in the Supplementary Materials).

### 2.5. Statistical Analysis

All quantitative data are expressed as the mean ± SD. The normality of the data was assessed by the Shapiro–Wilk test, followed by a two-tailed Student’s *t*-test or the Mann–Whitney U test. A *p*-value of < 0.05 was considered statistically significant. Statistical analyses were performed using SPSS software 22.0 (IBM Corp., Armonk, NY, USA) and Prism 8 software (GraphPad Software, San Diego, CA, USA).

## 3. Results

### 3.1. Radiochemical Synthesis and Quality Control

Each of the novel PSMA ligands was synthesized and purified by HPLC with a chemical purity of more than 98%, and MS analysis showed peaks of 991.99 (PSMA-4PY), 1042.50 (PSMA-Q), 1117.60 (PSMA-P), 1039.40 (PSMA-Q-1), 1025.39 (PSMA-Q-2), and 1073.39 (PSMA-Q-3) [M+H^+^] (Supplementary Figure S1). Each radiotracer was prepared with a radiochemical purity (RCP) of more than 95% and a molar activity (Am) of >20 GBq/μmol. The detailed results of quality control are shown in Table 1.

### 3.2. Partition Coefficient and Stability

**Figure 2:**
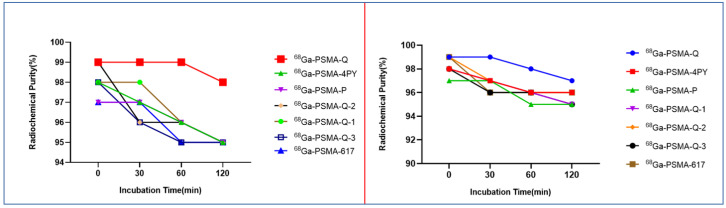
Stability of the novel tracers in PBS (**left**) and 5% BSA (**right**).

[^68^Ga]Ga-PSMA-4PY showed the highest hydrophilicity with, a log *p* value of −3.49 ± 0.01, followed by [^68^Ga]Ga-PSMA-Q (−3.24 ± 0.21), [^68^Ga]Ga-PSMA-Q-2 (3.14 ± 0.01), [^68^Ga]Ga-PSMA-Q-1 (−3.09 ± 0.19), [^68^Ga]Ga-PSMA-Q-3 (−2.99 ± 0.33), and [^68^Ga]Ga-PSMA-P (−2.77 ± 0.05). The hydrophilicity of all novel tracers was higher than that of [^68^Ga]Ga-PSMA-617 (log *p* = −2.54 ± 0.13). In addition, the hydrophilicity of these novel radiotracers was also compared with their retention times on radio-HPLC when the same mobile phase of 25/75 (*v*/*v*) acetonitrile/water containing 0.4% phosphoric acid at 1 mL/min was used. After incubation in PBS or 5% BSA at 37 °C for 2 h, the tracers were all stable in the two systems within the time tested (shown in Figure 2).

### 3.3. Determination of the PSMA Inhibition Constant

**Figure 3:**
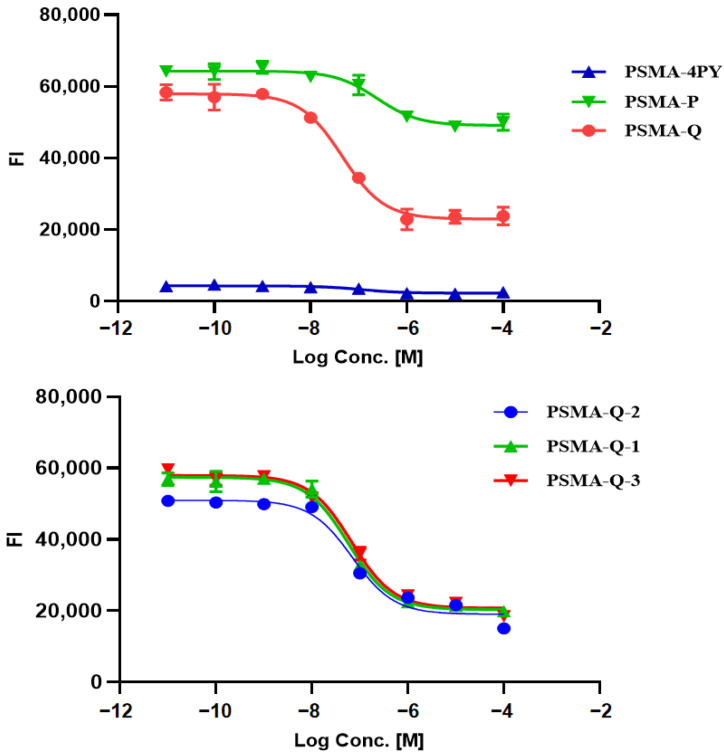
Determination of the binding affinity of novel PSMA ligands by enzyme-based assay (FI = fluorescence intensity).

The PSMA-inhibitory activity of novel PSMA ligands was analyzed by performing an enzyme-based assay on rhPSMA [11]. PSMA-Q showed the highest binding affinity to PSMA, with a Ki value of 8.11 ± 0.49 nM, which was lower than that of PSMA-617 (2.89 ± 1.07 nM, *p* < 0.05), followed by PSMA-Q-1 (10.21 ± 3.51 nM), PSMA-Q-3 (11.84 ± 4.29 nM), PSMA-Q-2 (12.26 ± 3.13 nM), PSMA-4PY (20.0 ± 0.87 nM), and PSMA-P (42.40 ± 2.11 nM) (as shown in Figure 3).

### 3.4. In Vitro Cellular Studies

**Figure 4:**
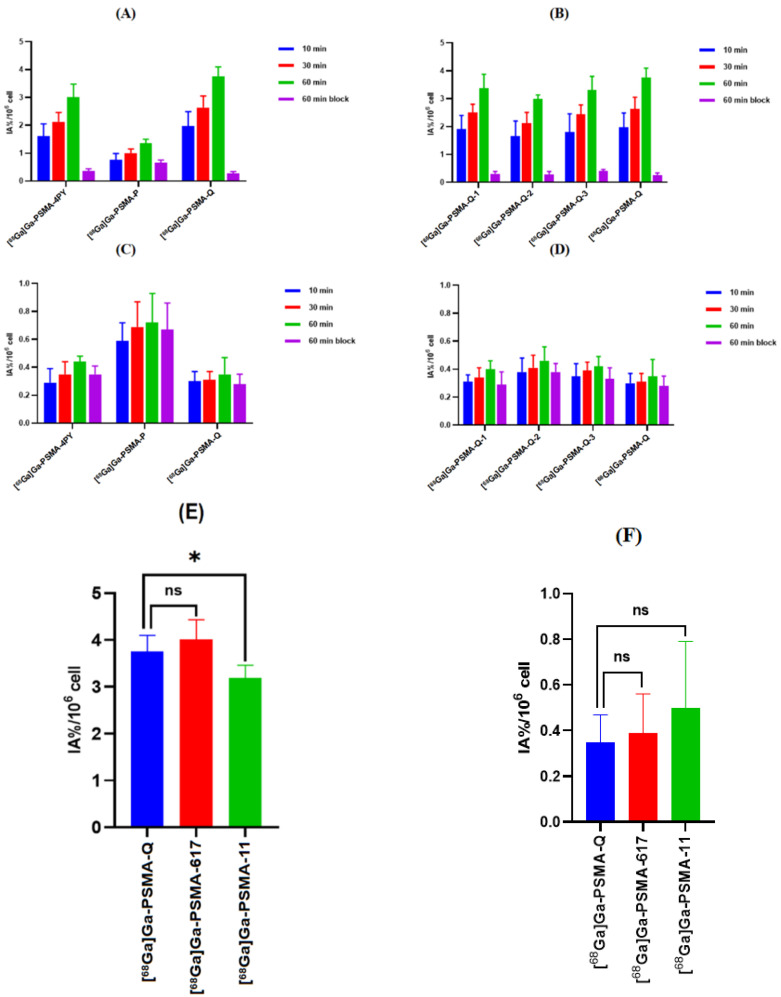
(**A**,**B**) Cell uptake of novel radiotracers in 22Rv1 cells. (**C**,**D**) Cell uptake of novel radiotracers in PC-3 cells. (**E**) Comparison of cell uptake of [^68^Ga]Ga-PSMA-Q, [^68^Ga]Ga-PSMA-11, and [^68^Ga]Ga-PSMA-617 in 22Rv1 cells at 60 min. (**F**) Comparison of cell uptake of [^68^Ga]Ga-PSMA-Q, [^68^Ga]Ga-PSMA-11, and [^68^Ga]Ga-PSMA-617 in PC-3 cells at 60 min p.i. (* *p* < 0.05).

Cell lines were kindly provided by the stem cell bank of the Chinese Academy of Sciences. The human prostate cancer epithelial cell lines 22Rv1 (mild PSMA+) and PC-3 (with no PSMA receptor) were used to evaluate the specific cell-binding affinity of the radiotracers, and the results were expressed as percentages of injected activity, IA%/10^6^. As shown in Figure 4, all of the novel tracers revealed substantially higher uptake in 22Rv1 cells than in PC-3 cells at all incubation time points (*p* < 0.01), among which [^68^Ga]Ga-PSMA-Q showed the highest uptake in 22Rv1 cells (3.75 ± 0.35 IA%/10^6^ at 60 min), followed by [^68^Ga]Ga-PSMA-Q-1 (3.38 ± 0.50 IA%/10^6^), [^68^Ga]Ga-PSMA-Q-3 (3.31 ± 0.49 IA%/10^6^), [^68^Ga]Ga-PSMA-4PY (3.01 ± 0.47 IA%/10^6^), and [^68^Ga]Ga-PSMA-Q-2 (2.99 ± 0.15 IA%/10^6^); [^68^Ga]Ga-PSMA-P showed the lowest uptake in 22Rv1 cells (1.35 ± 0.15 IA%/10^6^). The uptake in 22Rv1 cells was significantly blocked by co-incubation with 2-PMPA [24]. However, the uptake of each novel tracer in PC-3 cells (lower than 1 IA%/10^6^ at 60 min) was much lower than that in 22Rv1 cells (*p* < 0.05), and could not be blocked by co-incubation with 2-PMPA, indicating that the low-level uptake in PC-3 cells was nonspecific. In comparison, the uptake of [^68^Ga]Ga-PSMA-617 in 22Rv1 cells was 4.01 ± 0.42 IA%/10^6^ at 60 min, which was similar to that of [^68^Ga]Ga-PSMA-Q (*p* > 0.05), while the uptake of [^68^Ga]Ga-PSMA-11 in 22Rv1 cells was significantly lower than that of [^68^Ga]Ga-PSMA-Q (3.19 ± 0.27 IA%/10^6^ vs. 3.75 ± 0.35 IA%/10^6^, *p* < 0.05).

### 3.5. Pharmacokinetics

The blood pharmacokinetics of novel radiotracers and [^68^Ga]Ga-PSMA-617 in male ICR mice confirmed the two-compartment model according to the time–activity curves (as shown in Figure S3). The elimination half-lives were 15.15 ± 0.32 ([^68^Ga]Ga-PSMA-4PY), 17.86 ± 1.23 ([^68^Ga]Ga-PSMA-Q), 20.22 ± 0.97 ([^68^Ga]Ga-PSMA-Q-2), 21.17 ± 2.01 ([^68^Ga]Ga-PSMA-Q-1), 25.72 ± 1.87 ([^68^Ga]Ga-PSMA-Q-3), and 35.97 ± 4.42 ([^68^Ga]Ga-PSMA-P) min. The elimination of [^68^Ga]Ga-PSMA-4PY and [^68^Ga]Ga-PSMA-Q was similar to that of [^68^Ga]Ga-PSMA-11 (16.69 ± 1.77 min, *p* > 0.05), and much faster than that of [^68^Ga]Ga-PSMA-617 (28.46 ± 1.64 min, *p* < 0.05).

### 3.6. Biodistribution

**Figure 5:**
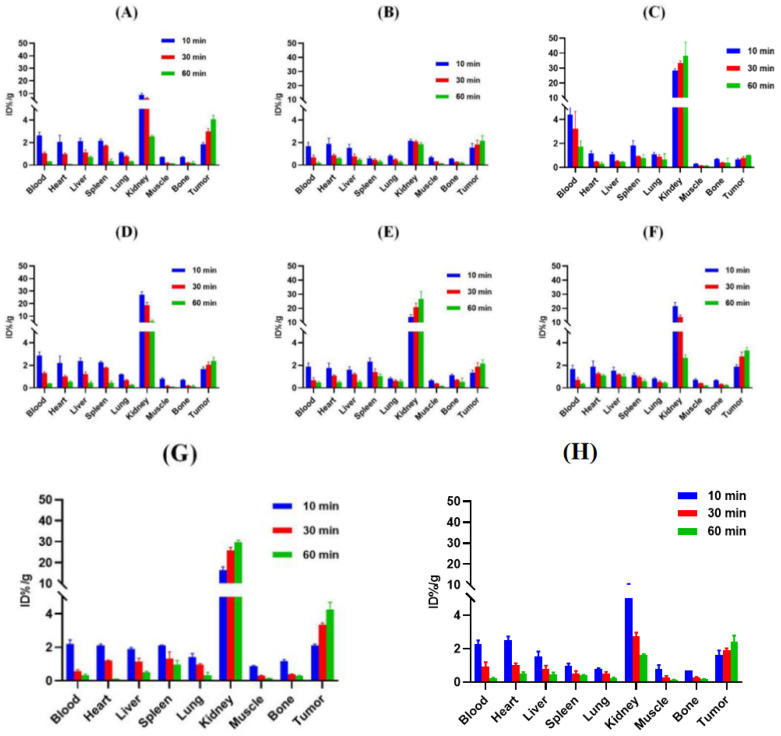
Uptake values for ex vivo tissues of (**A**) [^68^Ga]Ga-PSMA-Q, (**B**) [^68^Ga]Ga-PSMA-4PY, (**C**) [^68^Ga]Ga-PSMA-P, (**D**) [^68^Ga]Ga-PSMA-Q-1, (**E**) [^68^Ga]Ga-PSMA-Q-2, (**F**) [^68^Ga]Ga-PSMA-Q-3, (**G**) [^68^Ga]Ga-PSMA-617, and (**H**) [^68^Ga]Ga-PSMA-11 in 22Rv1 tumor-bearing mice (*n* = 3/time point).

**Figure 6:**
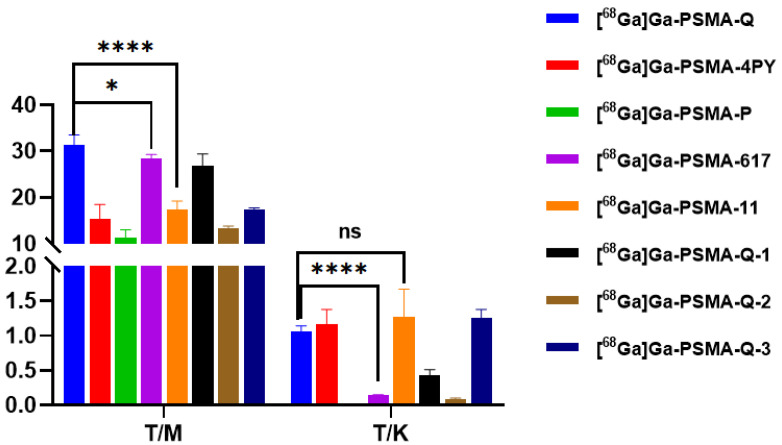
Tumor-to-muscle ratio (T/M) and tumor-to-kidney ratio (T/K) values of novel radiotracers and [^68^Ga]Ga-PSMA-617 (0.74 MBq/mouse) in 22Rv1 tumor-bearing mice at 60 min p.i. (*n* = 4/time point) (* *p* < 0.05, **** *p* < 0.0001).

Male BALB/c nude mice were used for the tumor models established. As shown in Figure 5 and Figure 6 and Tables S1–S8, the uptake of all tracers in 22Rv1 tumors increased within 60 min. [^68^Ga]Ga-PSMA-Q showed the highest uptake in tumors at 60 min p.i. (4.06 ± 0.36 ID%/g), which was similar to that of [^68^Ga]Ga-PSMA-617 (4.27 ± 0.10 ID%/g, *p* > 0.05), followed by [^68^Ga]Ga-PSMA-Q-3 (3.31 ± 0.29 ID%/g), [^68^Ga]Ga-PSMA-Q-1 (2.38 ± 0.31 ID%/g), [^68^Ga]Ga-PSMA-Q-4PY (2.15 ± 0.46 ID%/g), and [^68^Ga]Ga-PSMA-Q-2 (2.15 ± 0.33 ID%/g); [^68^Ga]Ga-PSMA-P showed the lowest uptake in tumors (1.01 ± 0.02 ID%/g). [^68^Ga]Ga-PSMA-Q exhibited the highest tumor-to-muscle (T/M) ratio (31.44 ± 2.09 at 60 min p.i.), which was higher than those of [^68^Ga]Ga-PSMA-617 (28.43 ± 0.84, *p* < 0.05), [^68^Ga]Ga-PSMA-11 (17.41 ± 1.80, *p* < 0.05), and all other novel tracers. Due to the high uptake in tumors and the fast clearance rate, the tumor-to-kidney (T/K) ratio of [^68^Ga]Ga-PSMA-Q (1.60 ± 0.08) was higher than those of [^68^Ga]Ga-PSMA-617 (0.14 ± 0.01, *p* < 0.05) and the other novel tracers.

In normal organs, the highest uptake of each tracer was observed in the kidneys. However, the clearance profiles were different between these tracers. [^68^Ga]Ga-PSMA-P, [^68^Ga]Ga-PSMA-Q-2, and [^68^Ga]Ga-PSMA-617 continuously accumulated in the kidneys within 60 min p.i.; the uptake of [^68^Ga]Ga-PSMA-4PY remained relatively stable, while [^68^Ga]Ga-PSMA-Q, [^68^Ga]Ga-PSMA-Q-1, and [^68^Ga]Ga-PSMA-Q-3 were washed out rapidly from the kidneys, and the uptake of [^68^Ga]Ga-PSMA-Q was only 2.53 ± 0.10 ID%/g at 60 min p.i. Other normal organs showed very low radioactivity accumulation and fast clearance.

### 3.7. Micro-PET Imaging

**Figure 7:**
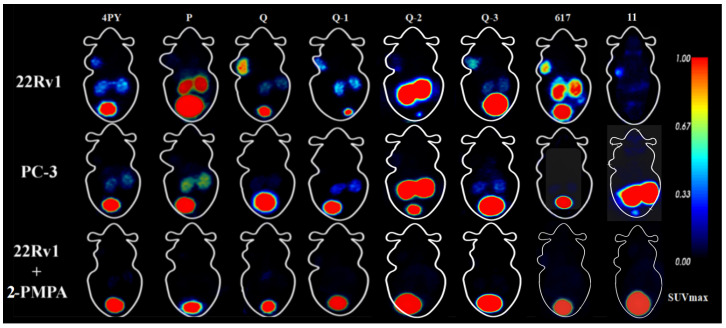
Micro-PET MIP images of novel tracers, [^68^Ga]Ga-PSMA-617, and [^68^Ga]Ga-PSMA-11 in 22Rv1 and PC-3 tumor-bearing mice at 60 min p.i..

**Figure 8:**
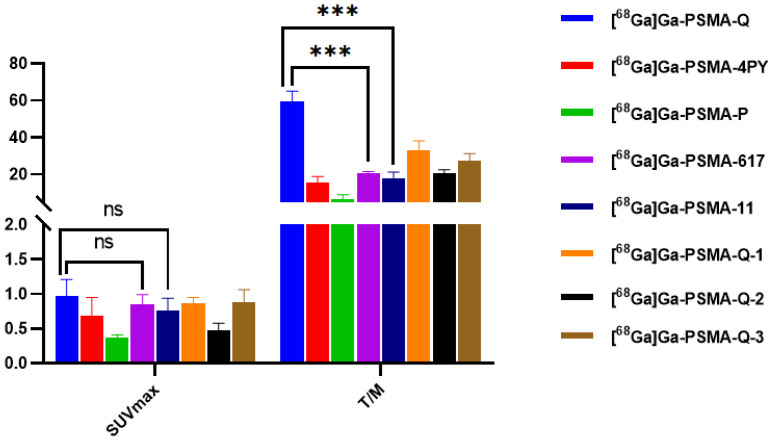
SUVmax of tumors and T/M values of novel tracers as well as [^68^Ga]Ga-PSMA-617 and [^68^Ga]Ga-PSMA-11 in 22Rv1 tumor-bearing mice at 60 min p.i. (*** *p* < 0.001).

Maximum-intensity projection images of all of the novel tracers as well as [^68^Ga]Ga-PSMA-617 and [^68^Ga]Ga-PSMA-11 in male 22Rv1 and PC-3 tumor-bearing mice at 60 min p.i. are shown in Figure 7. In tumor-bearing mice, different levels of radioactivity uptake and retention of novel tracers were observed in 22Rv1 tumors, while PC-3 tumors could not be visualized. The kidneys and bladder could also be visualized clearly, but due to the fast clearance rate, the uptake of [^68^Ga]Ga-PSMA-Q and [^68^Ga]Ga-PSMA-4PY in the kidneys was fairly low at 60 min p.i.. Very little radioactivity was found in other normal tissues. The results were consistent with our biodistribution data. The highest tumor uptake was observed when [^68^Ga]Ga-PSMA-Q was administered, with an SUVmax of 0.97 ± 0.24. The SUVmax of [^68^Ga]Ga-PSMA-Q in tumors was higher than those of [^68^Ga]Ga-PSMA-617 (0.85 ± 0.14, *p* = 0.42) and [^68^Ga]Ga-PSMA-11 (0.76 ± 0.18, *p* = 0.29), with no significant difference, while the tumor-to-muscle ratio of [^68^Ga]Ga-PSMA-Q was significantly higher than those of [^68^Ga]Ga-PSMA-617 (59.33 ± 5.72 vs. 20.43 ± 1.06, *p* < 0.05) and [^68^Ga]Ga-PSMA-11 (59.33 ± 5.72 vs. 17.95 ± 3.35, *p* < 0.05), making the tumor more clearly visualized in a clean background. The tumor uptakes of [^68^Ga]Ga-PSMA-Q-1 (SUVmax = 0.86 ± 0.09), [^68^Ga]Ga-PSMA-Q-3 (SUVmax = 0.88 ± 0.18), and [^68^Ga]Ga-PSMA-4PY (SUVmax = 0.69 ± 0.26) were slightly lower than those of [^68^Ga]Ga-PSMA-Q and [^68^Ga]Ga-PSMA-617, with no significant differences, but the tumor-to-muscle ratio for both of them (32.94 ± 4.56 for [^68^Ga]Ga-PSMA-Q-1 and 27.20 ± 9.80 for [^68^Ga]Ga-PSMA-Q-3) was significantly lower than that of [^68^Ga]Ga-PSMA-Q. [^68^Ga]Ga-PSMA-Q-2 (SUVmax = 0.47 ± 0.11) and [^68^Ga]Ga-PSMA-P (SUVmax = 0.37 ± 0.04) showed the lowest uptake in 22Rv1 tumors among all of the tracers (shown in Figure 8).

### 3.8. Radiotoxicity Study of [^68^Ga]Ga-PSMA-Q

**Figure 9:**
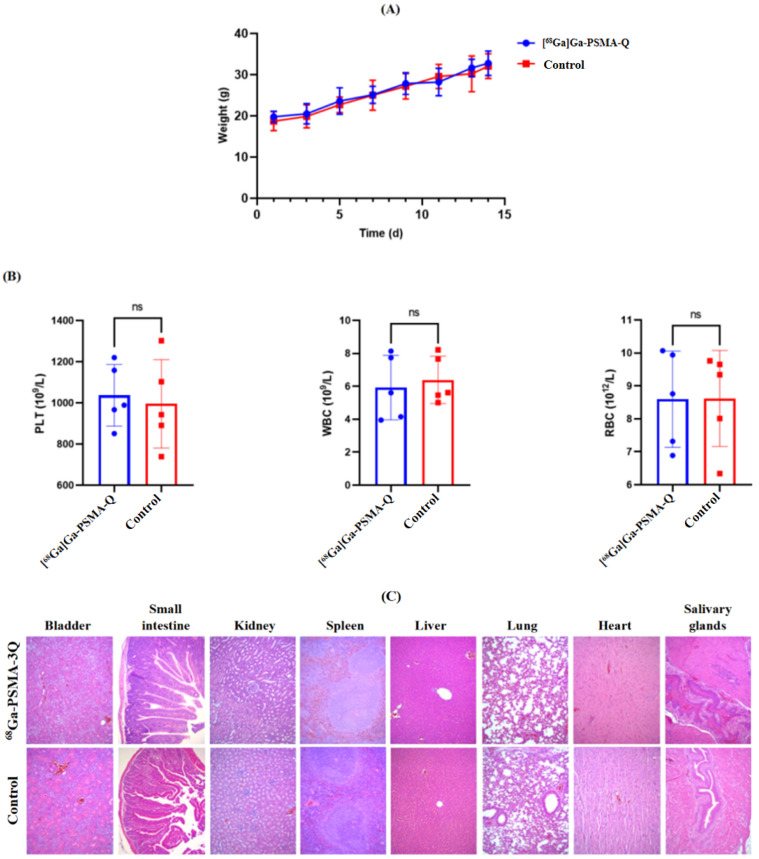
Radiotoxicity evaluation of [^68^Ga]Ga-PSMA-Q (37 MBq/mouse) in ICR mice (*n* = 5/group). (**A**) The weight changes of mice injected with [^68^Ga]Ga-PSMA-Q or saline (control) in the radiotoxicity study period. (**B**) Results of routine blood tests of mice in the [^68^Ga]Ga-PSMA-Q and control groups. (**C**) Results of H&E staining of the main organs (i.e., kidneys, heart, liver, spleen, salivary glands, small intestine, bladder, lungs) of mice in the [^68^Ga]Ga-PSMA-Q and control groups (WBC: white blood cell count, PLT: platelet count, RBC: red blood cell count. Values are expressed as the mean ± SD; *n* = 10; ns: not statistically significant).

The male ICR mice injected with [^68^Ga]Ga-PSMA-Q (37 MBq in 150 μL) or saline (150 μL) did not die within 14 days. There were no significant differences in body weight between the PSMA-Q and control groups. There were also no significant differences in the results of routine blood tests and H&E staining of the main organs between the experimental and control groups (shown in Figure 9).

## 4. Discussion

Noninvasive accurate diagnosis and precise treatment at the molecular level have become the most concerning scientific problems in recent years, and have attracted extensive attention and in-depth research. PET/CT is one molecular imaging tool with the aid of radionuclides that provides both functional and anatomical imaging simultaneously, making it a powerful means of dynamically and quantitatively observing physiological and biochemical changes in vivo [25,26,27]. Furthermore, a variety of benign and malignant diseases can be diagnosed by PET/CT imaging. This approach has been increasingly used in the clinic, and has shown great potential. ^18^F-FDG is the most frequently used PET tracer. The majority of malignant tumors are characterized by high glycolysis levels, which are used for ^18^F-FDG PET/CT imaging. The emergence of ^18^F-FDG has greatly improved the detection rate of a variety of malignant tumors—especially those in the early stages. However, ^18^F-FDG is not a perfect tumor imaging tracer [28]. First, it is not a tumor-specific tracer; second, it exhibits a poor imaging effect on some tumors with slow growth and low glucose utilization, including prostate cancer, and is prone to false negative results [29]. Therefore, the development of new specific molecular tracers can improve the accurate diagnosis and staging of tumors to a certain extent to realize the individualized treatment of patients.

Tumor-targeted compounds radiolabeled with radionuclides make it possible to image tumors specifically. Prostate-specific membrane antigen (PSMA)—an antigen overexpressed on almost all kinds of prostate cancer cell membranes—is considered to be an ideal target for the diagnosis and treatment of prostate cancer [29,30,31]. Small molecular agents based on glutamate urea showed folate hydrolase I activity and competitively inhibited the activity of NAALADase of PSMA, laying the foundation for high-affinity binding to PSMA expressed on prostate cancer cells [32,33]. In the last two decades, numerous small-molecule PSMA PET tracers have been developed and popularized in the clinic, such as [^68^Ga]Ga-PSMA-11, [^68^Ga]Ga-PSMA-617, [^68^Ga]Ga-PSMA-I&T, and ^18^F-DCFPyL [11,12,13,14,17,20], among which [^68^Ga]Ga-PSMA-11 and ^18^F-DCFPyL were approved by the U.S. FDA in the last two years.

However, all of the PSMA-targeted tracers mentioned above have their own limitations. The ^18^F-label tracer, ^18^F-DCFPyL, shows an outstanding image quality and diagnostic efficacy for PCa, but the technology and cost difficulties for ^18^F-DCFPyL production limit its use to some extent in many hospitals. [^68^Ga]Ga-PSMA-11, the first PSMA PET tracer for imaging approved by the FDA, despite having proven efficacy in the diagnosis of PCa, cannot be used in theranostic radiopharmaceuticals due to its structural limitations, posing an urgent clinical need. Meanwhile, in terms of PSMA-617, which can be labeled with both ^68^Ga and ^177^Lu, its tumor-to-background ratio and clearance rate still need to be improved, due to its relatively low hydrophilicity and inherent pharmacokinetic characteristics, which are crucial to PCa therapy. Therefore, further optimization of the PSMA ligands is still necessary.

To optimize the characteristics—including the pharmacokinetics—of PSMA-617, in this study, two steps of modification were adopted: first, the naphthyl group in PSMA-617 was replaced with three different groups, and in vitro and in vivo preclinical experiments were conducted to evaluate the three novel radiotracers 4-pyridyl (4PY), 3-quinoline (Q), and pyrene (P).

The purity of these three novel PSMA ligands was greater than 95%. According to the NAALADase assay, the PSMA-affinity-related Ki values for the novel agents were all less than 50 nM, similar to those of PSMA-617, PSMA-11, and other PSMA agents reported in the literature [11,34]. Hydrophilicity was greatly improved after modification with [^68^Ga]Ga-PSMA-617, especially for [^68^Ga]Ga-PSMA-4PY and [^68^Ga]Ga-PSMA-Q, with log *p* values of −3.49 ± 0.01 and −3.24 ± 0.21, respectively. The improvement in hydrophilicity also significantly accelerated the clearance rates of tracers in vivo. The elimination half-lives were 15.15 ± 0.32 and 17.86 ± 1.23 min for [^68^Ga]Ga-PSMA-4PY and [^68^Ga]Ga-PSMA-Q, respectively, which were much faster than that of [^68^Ga]Ga-PSMA-617, and similar to that of [^68^Ga]Ga-PSMA-11—a tracer characterized by rapid clearance. Due to the high uptake in 22Rv1 tumors and high hydrophilicity, along with rapid elimination from blood and normal organs—including the kidneys—the tumor-to-muscle and tumor-to-kidney ratios of [^68^Ga]Ga-PSMA-Q were both higher than those of [^68^Ga]Ga-PSMA-617, [^68^Ga]Ga-PSMA-11, and the other novel tracers, making the tumor easier to detect from the blood pool, and potentially aiding in the detection of tumors close to the kidneys. Moreover, fast clearance reduces the toxicity of radiopharmaceuticals in the blood and kidneys, offering a great advantage when used in radiopharmaceutical therapy. In the toxicity experiments in mice, no acute radiotoxicity-related adverse events were observed, indicating the good safety of [^68^Ga]Ga-PSMA-Q.

The 22Rv1 (PSMA +) and PC-3 (PSMA −) cells were selected according to the expression level of PSMA, and were used for in vitro cell-binding experiments and tumor model establishment [35,36]. As shown in the cell experiments, three novel radiotracers and [^68^Ga]Ga-PSMA-617 accumulated in 22Rv1 cells to varying degrees. [^68^Ga]Ga-PSMA-Q exhibited the highest uptake in 22Rv1 cells, which was comparable to that of [^68^Ga]Ga-PSMA-617 and significantly higher than that of [^68^Ga]Ga-PSMA-11, with no significant difference. The uptake of [^68^Ga]Ga-PSMA-P was the lowest, and a relatively high nonspecific uptake was observed in the blocking experiment. The accumulation of all tracers in our study was very low, and most of them could not be inhibited by 2-PMPA. The uptake of [^68^Ga]Ga-PSMA-P in PC-3 cells was slightly higher than that of the other tracers, confirming again that its nonspecific uptake value was higher.

Based on the results of the cell experiments, we further explored the in vitro biodistribution and micro-PET imaging of three radiotracers in 22Rv1 and PC-3 tumor-bearing mice, and the results were compared with those of [^68^Ga]Ga-PSMA-617. The radiotracers accumulated rapidly in 22Rv1 tumors post-injection. The uptake of [^68^Ga]Ga-PSMA-Q was also the highest among the novel tracers, being similar to that of [^68^Ga]Ga-PSMA-617, followed by [^68^Ga]Ga-PSMA-11, [^68^Ga]Ga-PSMA-4PY, and [^68^Ga]Ga-PSMA-P. All of the tracers accumulated very little in PC-3 tumors. The micro-PET results were generally consistent with the biodistribution profiles. In most normal tissues and organs, the uptake of radiotracers—including [^68^Ga]Ga-PSMA-617 and [^68^Ga]Ga-PSMA-11—was at a low level and eliminated rapidly. With the passage of time post-injection, the 22Rv1 tumor-to-background ratio was increased, which was conducive to detecting 22Rv1 tumors in micro-PET images. The considerably high uptake in the kidneys was caused by both PSMA expression in the kidneys and the excretion of radiotracers from the kidneys. The SUVmax of [^68^Ga]Ga-PSMA-Q and [^68^Ga]Ga-PSMA-4PY in 22Rv1 tumors was equivalent to that of [^68^Ga]Ga-PSMA-617 and [^68^Ga]Ga-PSMA-11, and there was no significant difference (*p* > 0.05). [^68^Ga]Ga-PSMA-Q showed the highest 22Rv1 tumor-to-muscle ratio among all of the novel radiotracers—greater than that of either [^68^Ga]Ga-PSMA-617 or [^68^Ga]Ga-PSMA-11 (*p* < 0.05). Meanwhile, [^68^Ga]Ga-PSMA-P showed a poor imaging effect on 22Rv1 tumors, making it difficult to distinguish between tumor and background tissues.

It could be seen from the animal experimental results that the results of micro-PET imaging were consistent with the in vitro biodistribution data, but there was no one-to-one correspondence between these data and the Ki values of the PSMA ligands. In vitro experiments preliminarily evaluated the affinity of agents for PSMA to some extent, and the results were quite different from the in vivo results. The final imaging effects of the radiotracers were not only determined by the affinity of agents, but also affected by many other factors, such as the hydrophilicity of the final products and their metabolism in vivo.

Based on the results discussed above, the novel radiotracer [^68^Ga]Ga-PSMA-Q, which contains a quinoline ring, was selected, and further modification was carried out.

As the most commonly used linker group, the cyclohexyl in [^68^Ga]Ga-PSMA-Q was then replaced with propyl, butyl, and phenyl in the second step, leading to the development of three other novel tracers: [^68^Ga]Ga-PSMA-Q-1, [^68^Ga]Ga-PSMA-Q-2, and [^68^Ga]Ga-PSMA-Q-3. Then, the same experiments as in the first part were conducted.

The hydrophilicity was decreased after the substitution of cyclohexyl, especially when benzylamine was used as a linker group. The log *p* value was decreased to −2.99 ± 0.33, and the elimination half-life of [^68^Ga]Ga-PSMA-Q-3 was prolonged accordingly (17.86 min for [^68^Ga]Ga-PSMA-Q vs. 25.72 min for [^68^Ga]Ga-PSMA-Q-3). The in vitro and in vivo studies showed that the highest uptake by 22Rv1 cells and tumors was obtained when cyclohexyl was used as the linker group. All of the results showed that the cyclohexyl linker group was still the most suitable.

The selected novel radiotracer [^68^Ga]Ga-PSMA-Q exhibited fairly high accumulation in PSMA+ tumors and satisfying pharmacokinetic characteristics, resulting in great potential for PCa tumor detection. Meanwhile, the precursor of PSMA-Q could be further radiolabeled with ^177^Lu for PCa treatment, due to its favorable tumor-to-background ratio. We have already conducted this work, and the results will be shown in the future.

## 5. Conclusions

Specific PSMA-targeted PET tracers play an important role in the diagnosis of prostate cancer. In this study, six novel PSMA radiotracers were developed and evaluated both in vitro and in vivo. [^68^Ga]Ga-PSMA-Q was ultimately selected due to its satisfying biodistribution and pharmacokinetic characteristics and its excellent imaging effect for PSMA (+) tumors, which were even better than those of [^68^Ga]Ga-PSMA-617 and [^68^Ga]Ga-PSMA-11. High specificity and detection rates for PCa lesions were exhibited in [^68^Ga]Ga-PSMA-Q PET/CT, thus demonstrating its great potential in the detection of prostate cancer.

## Figures and Tables

**Table 1:** Analytical data of the prepared radiotracers.

Tracer	RCP ^a^ (%)	Am (GBq/μmol)	Log *p*_pH = 7.4_ (*n* = 3)	Tr ^b^ (min)
[^68^Ga]Ga-PSMA-Q	>95%	29.33 ± 8.01	−3.24 ± 0.21	5.27
[^68^Ga]Ga-PSMA-4PY	>95%	23.09 ± 6.14	−3.49 ± 0.01	4.36
[^68^Ga]Ga-PSMA-P	>95%	21.59 ± 4.14	−2.77 ± 0.05	8.46
[^68^Ga]Ga-PSMA-Q-1	>95%	26.87 ± 9.00	−3.09 ± 0.19	3.75
[^68^Ga]Ga-PSMA-Q-2	>95%	29.75 ± 7.56	−3.14 ± 0.01	1.72
[^68^Ga]Ga-PSMA-Q-3	>95%	27.19 ± 8.89	−2.99 ± 0.33	9.75
[^68^Ga]Ga-PSMA-617	>95%	22.42 ± 5.23	−2.54 ± 0.13	10.14

^a^ RCP was detected by radio-HPLC. ^b^ Retention time of radiotracers on analytical HPLC using 25% acetonitrile mixed with 0.4% phosphoric acid as the mobile phase, at a flow rate of 1 mL/min.

## Data Availability

All of the data presented are available in the manuscript and the Supplementary Materials.

## Supplementary Materials

The following supporting information can be downloaded at www.mdpi.com/article/10.3390/ph15081001/s1: General experimental procedures; MS of synthesized precursors; radiosynthesis of novel radiotracers and quality control; partition coefficient; stability and cell binding affinity; pharmacokinetics; biodistribution; and micro-PET imaging of novel radiotracers.
