# Supplementary material for: Optimized 68Ga-Labeled Urea-Based PSMA-Targeted PET Tracers for Prostate Cancer
Source: Pharmaceuticals (Basel). 2022 Aug 14;15(8):1001. doi: 10.3390/ph15081001 (PMC9414910; doi:10.3390/ph15081001)
Supplement: Supplementary file 1 [file pharmaceuticals-15-01001-s001.zip › pharmaceuticals-1693452-supplementary.pdf]

Supplementary Material

Synthesis of PSMA ligands PSMA-Q, PSMA-4PY and PSMA-P

All the ligands were synthesized via solid-phase synthesis which was performed according to Fmoc peptide synthesis protocol.

i) Fmoc-Glu-urea-Lys-resin (0.22 mmol/g, 100 mg) was added into the solid phase synthesis tube, the compound was rinsed with DCM (3 × 5 min × 2 mL) and DMF (3 × 5 min × 2 mL). To remove Fmoc, the compound was rinsed with DMF containing 20% piperidine (1 × 2 min × 2 mL, 2 × 10 min × 2 mL) and DMF (6 × 1 min × 2 mL).

ii) A mixture of Fmoc-3-(3-quinoline)-D-alanine (Q) or Fmoc-3-(4-pyridyl)-D-alanine (4PY) or Fmoc-3-(pyrene)-D-alanine (P) (0.06 mmol, 26.2 mg), HBTU (0.072 mmol, 27 mg), HOBt (0.072 mmol, 10 mg), DIPEA (0.15 mmol, 25 μL) was stirred in DMF (3 mL) at room temperature for 15 min. The activated 2-pyridine-alanine was added into the cleaned resin and reacted under nitrogen for 1 hour and then rinsed with DMF (6 × 1 min × 2 mL). To remove Fmoc, the compound was rinsed with DMF containing 20% piperidine (1 × 2 min × 2 mL, 2 × 10 min × 2 mL) and DMF (6 × 1 min × 2 mL).

iii) A mixture of trans-4-(Fmoc-aminomethyl)-cyclohexanecarboxylic acid (3 M, 0.06 mmol, 23 mg), HBTU (0.072 mmol, 27 mg), HOBt (0.072 mmol, 10 mg), DIPEA (0.15 mmol, 25 μL) was stirred in 3 mL DMF at room temperature for 15 min. The activated cyclohexane formic acid was added into the cleaned

resin and reacted under nitrogen for 1 hour and then rinsed with DMF (6 × 1 min × 2 mL). To remove Fmoc, the compound was rinsed with DMF containing 20% piperidine (1 × 2 min × 2 mL, 2 × 10 min × 2 mL) and DMF (6 × 1 min × 2 mL).

iiii) A mixture of DOTA tert-butyl ester (3 M, 0.06 mmol, 34.4 mg), HBTU (0.072 mmol, 27 mg), HOBt (0.072 mmol, 10 mg) and DIPEA (0.15 mmol, 25 μL) was stirred in 3 mL of DMF at room temperature for 15 min. The activated DOTA tert-butyl ester was added to the cleaned resin and reacted under nitrogen for 1 hour and then rinsed with DMF (6 × 1 min × 2 mL). A solution of TFA (4.5 mL), TIPS (250 μL), H_2O (250 μL) was added and reacted at room temperature for 2 h. The filtrate was collected and washed with TFA (2 mL). The final products (PSMA-Q, PSMA-4PY and PSMA-P) were purified by HPLC.

Synthesis of PSMA ligands PSMA-Q-1, PSMA-Q-2 PSMA-Q-3

i) Fmoc-Glu-urea-Lys-resin (0.22 mmol/g, 100 mg) was added into the solid phase synthesis tube, the compound was rinsed with DCM (3 × 5 min × 2 mL) and DMF (3 × 5 min × 2 mL). To remove Fmoc, the compound was rinsed with DMF containing 20% piperidine (1 × 2 min × 2 mL, 2 × 10 min × 2 mL) and DMF (6 × 1 min × 2 mL).

ii) A mixture of Fmoc-3-(3-quinoline)-D-alanine (0.06 mmol, 26.2 mg), HBTU (0.072 mmol, 27 mg), HOBt (0.072 mmol, 10 mg), DIPEA (0.15 mmol, 25 μL)

was stirred in DMF (3 mL) at room temperature for 15 min. The activated 2-pyridine-alanine was added into the cleaned resin and reacted under nitrogen for 1 hour and then rinsed with DMF (6 × 1 min × 2 mL). To remove Fmoc, the compound was rinsed with DMF containing 20% piperidine (1 × 2 min × 2 mL, 2 × 10 min × 2 mL) and DMF (6 × 1 min × 2 mL).

iii) A mixture of trans-4-(Fmoc-aminomethyl)-butyric acid (PSMA-Q-1) or trans-4-(Fmoc-aminomethyl)-valeric acid (PSMA-Q-2) or trans-4-(Fmoc-aminomethyl)-benzoic acid (PSMA-Q-3) (3 M, 0.06 mmol, 23 mg), HBTU (0.072 mmol, 27 mg), HOBt (0.072 mmol, 10 mg), DIPEA (0.15 mmol, 25 μL) was stirred in 3 mL DMF at room temperature for 15 min. The activated butyric acid or valeric acid or benzoic acid was added into the cleaned resin and reacted under nitrogen for 1 hour and then rinsed with DMF (6 × 1 min × 2 mL). To remove Fmoc, the compound was rinsed with DMF containing 20% piperidine (1 × 2 min × 2 mL, 2 × 10 min × 2 mL) and DMF (6 × 1 min × 2 mL).

iiii) A mixture of DOTA tert-butyl ester (3 M, 0.06 mmol, 34.4 mg), HBTU (0.072 mmol, 27 mg), HOBt (0.072 mmol, 10 mg) and DIPEA (0.15 mmol, 25 μL) was stirred in 3 mL of DMF at room temperature for 15 min. The activated DOTA tert-butyl ester was added to the cleaned resin and reacted under nitrogen for 1 hour and then rinsed with DMF (6 × 1 min × 2 mL). A solution of TFA (4.5 mL), TIPS (250 μL), H_2O (250 μL) was added and reacted at room temperature for 2 h. The filtrate was collected and washed with TFA (2 mL).

The final products (PSMA-Q, PSMA-Q-1 and PSMA-Q-2 and PSMA-Q-3) were purified by HPLC.

Radiolabeling

HCl (0.05 M, 4 mL) was used to treat the generator (ITG $^{68}Ge/^{68}Ga$ generator) the day before labeling. $^{68}GaCl_3$ was obtained from $^{68}Ge/^{68}Ga$ generator with HCl (0.05 M, 2 mL) in the reactor, the precursor (30 μg) was dissolved in H_2O (30 μL), the sodium acetate (1 M, 130 μL) was added into the reaction tube in advance, and the mixture was reacted at 85 °C for 5 min, in which 10 mL water was added after cooling. Subsequently, the mixture was passed through the Vac C-18 Cartridge and the C-18 Cartridge was washed with H_2O (10 mL). The product was obtained by eluting the C-18 Cartridge with ethanol (0.5 mL), passing through a sterile filter, and diluting with saline (5 mL).

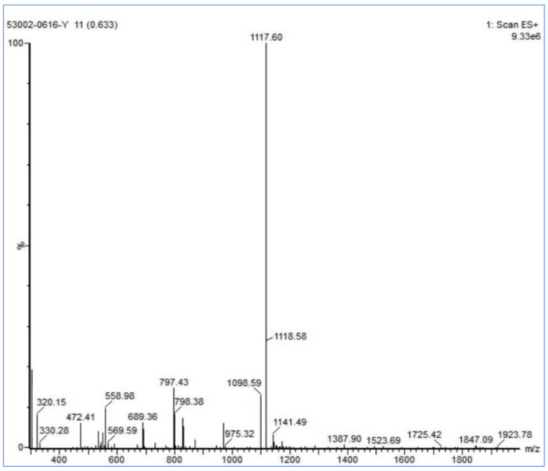

PSMA-4PY

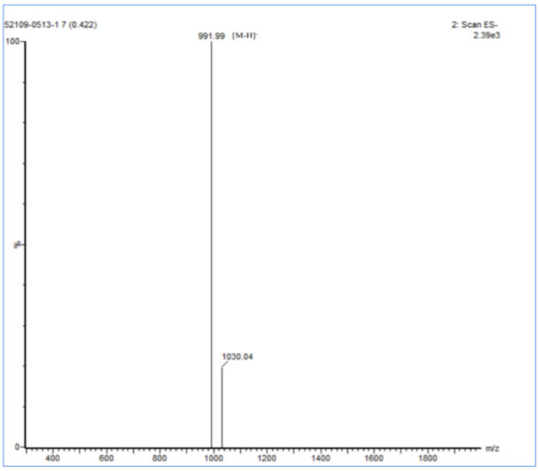

PSMA-P

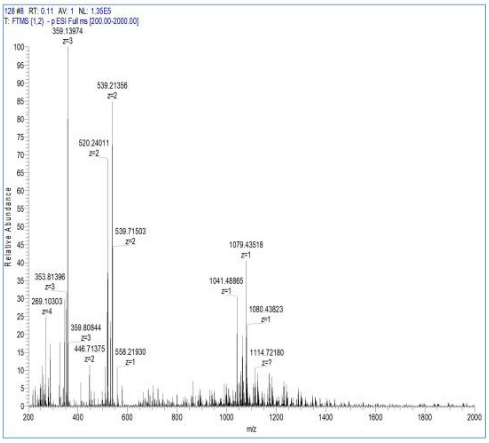

PSMA-Q

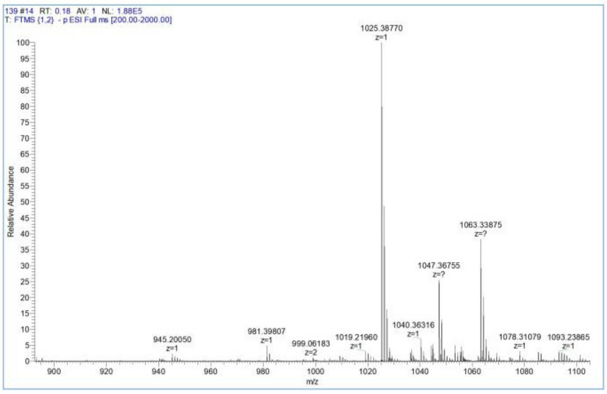

PSMA-Q-1

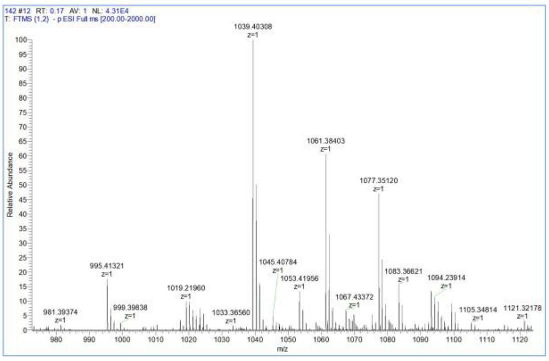

PSMA-Q-2

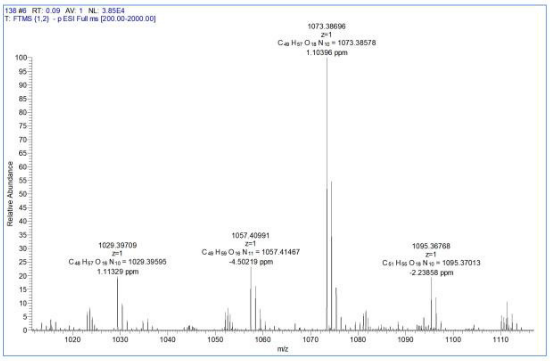

PSMA-Q-3

Figure S1. ESI-MS chromatogram of novel PSMA ligands.

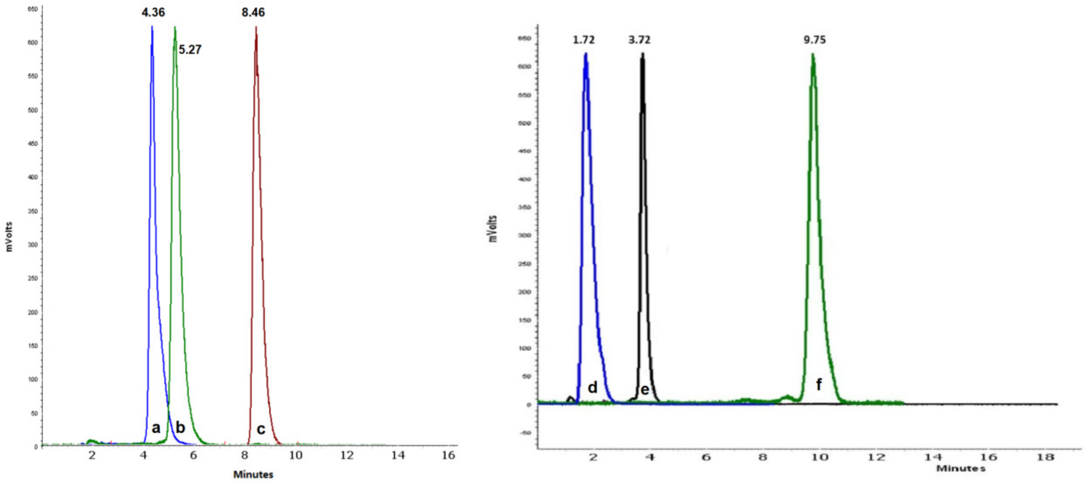

Figure S2. Analytical radio-HPLC chromatogram of novel radiotracers (a: [^{68}Ga]Ga-PSMA-4PY, b: [^{68}Ga]Ga-PSMA-Q, c: [^{68}Ga]Ga-PSMA-P; d: [^{68}Ga]Ga-PSMA-Q-2; e: [^{68}Ga]Ga-PSMA-Q-1; f: [^{68}Ga]Ga-PSMA-Q-3). The mobile phase was 25% acetonitrile mixed with 0.4% phosphoric acid at 1 mL/min.

Quality Control

All the tests of quality control were conducted before further studies. Radiochemical purity was determined by radio-HPLC using 10% acetonitrile and 90% water (containing 0.1% TFA) as the mobile phase. The specific activity was analyzed by HPLC via the UV calibration curve.

Partition Coefficient

Octanol-water partition coefficient was determined by adding novel tracers or [^{68}Ga]Ga-PSMA-617 or [^{68}Ga]Ga-PSMA-11 (0.37 MBq in 100 μL), 2.9 mL of phosphate-buffered saline (0.1 M, pH 7.4) and 3 mL of octanol into a tube. The mixture was vortexed (2,000 rpm × 2 min) and centrifuged (3,000 rpm × 5 min). Then, 3 samples (1 mL) from each phase were collected and the radioactivity was measured by a γ-counter respectively. The experiment was repeated 3 times. Log p value was calculated using log p = log (CPM for octanol/CPM for

PBS) and reported as log p ± SD.

In Vitro Stability

The in vitro stability of radiotracers was determined by measuring radiochemical purity (RCP) in saline and 5% bovine serum albumin (BSA) at 37 °C at different time intervals (30 min, 60 min, 120 min) using radio-HPLC.

NAALADase Assay

Recombinant human PSMA (rhPSMA, R&D systems, Wiesbaden, Germany) was diluted in assay buffer (50 mM HEPES, 0.1 M NaCl, pH 7.5) to 0.4 μg/mL. The substrate NAAG (Sigma, Taufkirchen, Germany, 160 μM final concentration, 125 μL) was mixed with PSMA-Q, PSMA-4PY, PSMA-P, PSMA-Q-1, PSMA-Q-2, PSMA-Q-3 (0.04 to 4000 nM, 125 μL), respectively. The mixture was combined with 125 μL of rhPSMA solution and incubated for 1 hour at 37 °C. The reaction was stopped by heating at 95 °C for 5 min. 250 μL of ortho-phthaldialdehyde solution (OPA, 15 mM, Sigma, Taufkirchen, Germany) was added to each vial and incubated for 3 min at room temperature. 200 μL aliquot of each vial was added into a 96 black well plate and read at excitation and emission wavelengths of 350 and 450 nm using a microplate reader. Assays were performed in triplicate. The data were analyzed by the one-site total binding regression algorithm of GraphPad Prism 8.0.

Cell Lines and Culture Condition

Cell lines were kindly provided by the stem cell bank, the Chinese Academy of Sciences. The human prostate cancer cell lines 22Rv1 (mild PSMA positive) and PC3 (PSMA negative) were cultured in RPMI 1640 medium or F12K medium (CORNING, America), supplemented with 10% Fetal Bovine Serum (GIBCO, The Netherlands) and 1% penicillin-streptomycin (CORNING, America) in a humidified incubator at 37 °C under 5% CO_2. Each cell line was passaged when it was at around 80-90% confluence, and harvested using trypsin-ethylenediaminetetraacetic acid (trypsin-EDTA; 0.25% trypsin, 0.02% EDTA, all from KeyGEN BioTECH).

In Vitro Cellar Studies

Cells were seeded separately in 24 well plates (1 x 10^5 cells in 1 mL medium/well) and incubated overnight, the medium was changed 2 h before the experiment. For the binding experiments, cells were incubated with novel radiotracers or [^{68}Ga]Ga-PSMA-617 (0.074 MBq/well in 100 μL) at 37 °C for 10 min, 30 min and 60 min (6 wells/time point). After incubation, the medium was removed and the cells were washed twice with ice-cold phosphate-buffered saline (0.5 mL). Finally, the cells were lysed by NaOH (0.5 M, 0.5 mL). Coincubation of 2-(phosphonomethyl) pentane-1,5-dioic acid

(2-PMPA) solution (2 μg/well) and each radiotracer for 60 min was conducted as the blocking experiment. The radioactivity of cells was measured by a γ-counter. The studies were performed in triplicate and the results were reported as the percentage injected activity (IA%/10^6 cells).

Pharmacokinetics

Injection of novel radiotracers or [^{68}Ga]Ga-PSMA-617 or [^{68}Ga]Ga-PSMA-11 (7.4 MBq in 150 μL) was administered intravenously via tail into ICR male mice (5 mice/group), and the blood sample (5 μL) of each mouse was collected by cutting down the tail end at different time points post injection. The radioactivity of blood samples were measured by a γ-counter and the pharmacokinetics data was calculated using the WinNonlin software.

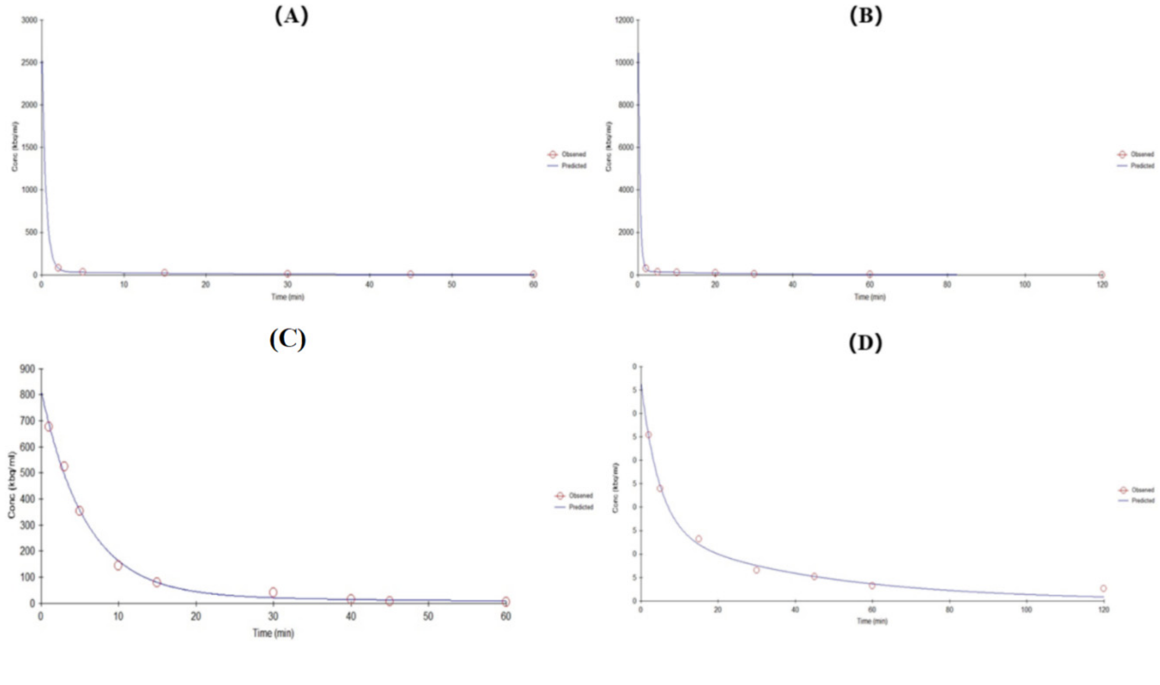

Figure S3. Time-activity curves of the blood samplesafter injection of 7.4 MBq of [^{68}Ga]Ga-PSMA-4PY (A), [^{68}Ga]Ga-PSMA-Q (B),[^{68}Ga]Ga-PSMA-P (C), [^{68}Ga]Ga-PSMA-617 (D), [^{68}Ga]Ga-PSMA-11 (E), [^{68}Ga]Ga-PSMA-Q-1 (F), [^{68}Ga]Ga-PSMA-Q-2 (G) and [^{68}Ga]Ga-PSMA-Q-3 (H).

Tumor Models

BALB/c nude mice (male, 4-6 weeks) were inoculated subcutaneously with 22Rv1 or PC-3 cells (1×10^7 cells/mL, 0.2 mL) on the left flank, when the tumors had grown up to

100 mm^3, the mice underwent biodistribution study and Micro-PET imaging.

Biodistribution

The 22Rv1 tumor-bearing mice were injected with each novel radiotracer or [^{68}Ga]Ga-PSMA-617 and [^{68}Ga]Ga-PSMA-11 (n = 9/group, n = 3/time point, 0.74 MBq in 150 μL per mouse). Mice were sacrificed at different time points p.i. (10 min, 30 min, 60 min), tumors and interested organs were harvested, weighed and measured by a γ-counter, the results were expressed as the percentage of injected dose per gram (ID%/g).

Table S1. Uptake values for ex vivo tissues of [^{68}Ga]Ga-PSMA-Q (0.74 MBq/mice) in 22Rv1 tumor-bearing mice (10, 30 and 60 min p.i., n = 3/time point).

	10 min		30 min		60 min	
Organ	Mean (ID%/g)	SD (ID%/g)	Mean (ID%/g)	SD (ID%/g)	Mean (ID%/g)	SD (ID%/g)
Blood	2.62	0.30	1.04	0.12	0.31	0.02
Heart	2.07	0.59	0.98	0.09	0.08	0.01
Liver	2.13	0.24	1.14	0.22	0.70	0.10
Spleen	2.16	0.15	1.73	0.06	0.35	0.19
Lung	1.10	0.07	0.76	0.08	0.33	0.03
Kidney	8.79	1.32	5.91	0.76	2.46	0.10
Muscle	0.70	0.03	0.21	0.01	0.13	0.02
Bone	0.72	0.04	0.22	0.01	0.21	0.14
Tumor	1.86	0.15	2.98	0.26	4.06	0.36

Table S2. Uptake values for ex vivo tissues of [^{68}Ga]Ga-PSMA-4PY (0.74 MBq/mice) in 22Rv1 tumor-bearing mice (10, 30 and 60 min p.i., n = 3/time point).

	10 min		30 min		60 min	
Organ	Mean (ID%/g)	SD (ID%/g)	Mean (ID%/g)	SD (ID%/g)	Mean (ID%/g)	SD (ID%/g)
Blood	1.68	0.32	0.68	0.21	0.21	0.08
Heart	1.87	0.52	0.86	0.11	0.58	0.07
Liver	1.53	0.30	0.78	0.21	0.47	0.11
Spleen	0.59	0.18	0.47	0.10	0.33	0.09
Lung	0.83	0.09	0.50	0.08	0.25	0.11
Kidney	2.15	0.15	2.08	0.10	1.85	0.17
Muscle	0.67	0.10	0.31	0.01	0.14	0.02
Bone	0.56	0.04	0.28	0.01	0.21	0.04
Tumor	1.56	0.35	1.83	0.38	2.15	0.46

Table S3. Uptake values for ex vivo tissues of [^{68}Ga]Ga-PSMA-P (0.74 MBq/mice) in 22Rv1 tumor-bearing mice (10, 30 and 60 min p.i., n = 3/time point).

	10 min		30 min		60 min	
Organ	Mean (ID%/g)	SD (ID%/g)	Mean (ID%/g)	SD (ID%/g)	Mean (ID%/g)	SD (ID%/g)
Blood	4.40	0.59	3.24	1.01	1.72	0.34
Heart	1.16	0.15	0.49	0.03	0.31	0.09
Liver	1.10	0.12	0.51	0.05	0.46	0.04
Spleen	1.85	0.29	0.91	0.04	0.77	0.23
Lung	1.10	0.11	0.90	0.11	0.65	0.34
Kidney	28.38	0.84	33.34	1.00	38.73	6.77
Muscle	0.28	0.03	0.15	0.02	0.10	0.04
Bone	0.69	0.03	0.40	0.02	0.36	0.25
Tumor	0.65	0.07	0.78	0.09	1.01	0.02

Table S4. Uptake values for ex vivo tissues of [^{68}Ga]Ga-PSMA-617 (0.74 MBq/mice) in 22Rv1 tumor-bearing mice (10, 30 and 60 min p.i., n = 3/time point).

	10 min		30 min		60 min	
Organ	Mean (ID%/g)	SD (ID%/g)	Mean (ID%/g)	SD (ID%/g)	Mean (ID%/g)	SD (ID%/g)
Blood	2.21	0.24	0.58	0.08	0.33	0.07
Heart	2.11	0.08	1.20	0.04	0.10	0.01
Liver	1.89	0.10	1.13	0.22	0.51	0.06
Spleen	2.11	0.02	1.31	0.42	0.95	0.25
Lung	1.40	0.23	0.95	0.07	0.33	0.17
Kidne	16.30	1.58	25.77	1.56	29.78	0.90

y						
Muscle	0.87	0.04	0.31	0.03	0.15	0.01
Bone	1.17	0.10	0.38	0.03	0.29	0.03
Tumor	2.10	0.09	3.36	0.10	4.27	0.41

Table S5. Uptake values for ex vivo tissues of [^{68}Ga]Ga-PSMA-11 (0.74 MBq/mice) in 22Rv1 tumor-bearing mice (10, 30 and 60 min p.i., n = 3/time point).

	10 min		30 min		60 min	
Organ	Mean (ID%/g)	SD (ID%/g)	Mean (ID%/g)	SD (ID%/g)	Mean (ID%/g)	SD (ID%/g)
Blood	2.28	0.22	0.91	0.28	0.22	0.05
Heart	2.54	0.19	1.01	0.12	0.53	0.07
Liver	1.53	0.30	0.78	0.21	0.47	0.11
Spleen	0.99	0.13	0.53	0.15	0.40	0.04
Lung	0.77	0.06	0.53	0.09	0.21	0.07
Kidney	10.20	0.33	2.76	0.21	1.61	0.07
Muscle	0.81	0.21	0.30	0.07	0.14	0.01
Bone	0.72	0.00	0.30	0.02	0.17	0.03
Tumor	1.61	0.29	1.90	0.12	2.38	0.36

Table S6. Uptake values for ex vivo tissues of [^{68}Ga]Ga-PSMA-Q-1 (0.74 MBq/mice) in 22Rv1 tumor-bearing mice (10, 30 and 60 min p.i., n = 3/time point).

	10 min		30 min		60 min	
Organ	Mean (ID%/g)	SD (ID%/g)	Mean (ID%/g)	SD (ID%/g)	Mean (ID%/g)	SD (ID%/g)
Blood	2.88	0.29	1.31	0.13	0.37	0.02
Heart	2.21	0.61	1.01	0.10	0.54	0.07
Liver	2.38	0.28	1.23	0.19	0.45	0.11
Spleen	2.26	0.11	1.79	0.06	0.45	0.13
Lung	1.19	0.04	0.68	0.06	0.26	0.06
Kidney	27.12	2.31	18.86	2.11	5.70	1.10
Muscle	0.81	0.09	0.18	0.04	0.09	0.02
Bone	0.72	0.04	0.21	0.01	0.15	0.09
Tumor	1.67	0.20	2.07	0.22	2.38	0.31

Table S7. Uptake values for ex vivo tissues of [^{68}Ga]Ga-PSMA-Q-2 (0.74 MBq/mice) in 22Rv1 tumor-bearing mice (10, 30 and 60 min p.i., n = 3/time point).

	10 min		30 min		60 min	
Organ	Mean (ID%/g)	SD (ID%/g)	Mean (ID%/g)	SD (ID%/g)	Mean (ID%/g)	SD (ID%/g)
Blood	1.87	0.34	0.69	0.22	0.47	0.08
Heart	1.76	0.44	1.08	0.09	0.50	0.10
Liver	1.62	0.27	1.21	0.10	0.52	0.15
Spleen	2.34	0.32	1.41	0.29	1.05	0.20
Lung	0.83	0.10	0.61	0.11	0.55	0.17
Kidney	13.88	1.56	20.74	2.86	26.62	5.33
Muscle	0.65	0.08	0.37	0.03	0.16	0.02
Bone	1.12	0.09	0.71	0.04	0.53	0.33
Tumor	1.34	0.21	1.87	0.36	2.15	0.33

Table S8. Uptake values for ex vivo tissues of [^{68}Ga]Ga-PSMA-Q-3 (0.74 MBq/mice) in 22Rv1 tumor-bearing mice (10, 30 and 60 min p.i., n = 3/time point).

	10 min		30 min		60 min	
Organ	Mean (ID%/g)	SD (ID%/g)	Mean (ID%/g)	SD (ID%/g)	Mean (ID%/g)	SD (ID%/g)
Blood	1.68	0.32	0.72	0.19	0.34	0.05
Heart	1.87	0.52	1.29	0.10	1.11	0.08
Liver	1.53	0.30	1.18	0.08	1.01	0.21
Spleen	1.12	0.18	0.94	0.11	0.59	0.18
Lung	0.83	0.09	0.53	0.10	0.43	0.09
Kidney	21.45	2.66	13.88	1.35	2.65	0.27
Muscle	0.72	0.09	0.40	0.03	0.19	0.02
Bone	0.67	0.06	0.33	0.03	0.23	0.03
Tumor	1.88	0.19	2.77	0.40	3.31	0.29

Table S9. Tumor-to-muscle (T/M) ratio and tumor-to-kidney (T/K) ratio of radiotracers at 60 min p.i.

Radiotracer	T/M	T/K
[^{68}Ga]Ga-PSMA-Q	31.44 ± 2.09	1.60 ± 0.08
[^{68}Ga]Ga-PSMA-4PY	15.43 ± 3.02	1.16 ± 0.22
[^{68}Ga]Ga-PSMA-P	11.31 ± 1.69	0.03 ± 0.00
[^{68}Ga]Ga-PSMA-Q-1	26.82 ± 2.58	0.43 ± 0.08
[^{68}Ga]Ga-PSMA-Q-2	13.41 ± 0.39	0.08 ± 0.02
[^{68}Ga]Ga-PSMA-Q-3	17.44 ± 0.31	1.25 ± 0.13
[^{68}Ga]Ga-PSMA-617	28.43 ± 0.84	0.14 ± 0.01
[^{68}Ga]Ga-PSMA-11	17.36 ± 1.66	1.51 ± 0.27

Micro-PET Imaging

The Mice bearing 22Rv1 and PC-3 (n = 3/group) xenograft tumors were intravenously injected with [^{68}Ga]Ga-PSMA-Q, [^{68}Ga]Ga-PSMA-4PY, [^{68}Ga]Ga-PSMA-P, [^{68}Ga]Ga-PSMA-Q-1, [^{68}Ga]Ga-PSMA-Q-2, [^{68}Ga]Ga-PSMA-Q-3 (7.4 MBq in 150 μL) via the tail vein, respectively. Co-injection with 2-PMPA (50 μg) and each novel tracer (7.4 MBq in 150 μL) was used for blocking. Then the mice were anesthetized with 3% (v/v) isoflurane and underwent Micro-PET scans with continuous 1% (v/v) isoflurane. The images were acquired at 60 min p.i.. In comparison, [^{68}Ga]Ga-PSMA-617 or [^{68}Ga]Ga-PSMA-11 (7.4 MBq in 150 μL) was injected into another group of mice (n = 3), and Micro-PET schedule was performed as described before.

Imaging was performed on a Super Nova PET/CT scanner using a 100-mm-transaxial field of view for PET and 110-mm for CT. Three-dimensional ordered-subsets expectation maximization reconstruction

algorithms with attenuation and random corrections were used for reconstruction. Finally, the images were displayed on Recon/Avator-s-10 workstation. The SUVmax for each ROI over tumor and muscle was measured.

Radiotoxicity Study of [^{68}Ga]Ga-PSMA-Q

[^{68}Ga]Ga-PSMA-Q (37 MBq in 150 μL) was injected into 5 ICR male mice, and another 5 were injected with 150 μL of saline as a comparison. At 14 d post injection, venous blood was drawn from the tail vein for routine blood tests, then the mice were weighed and euthanized, and the main organs were harvested and fixed in 4% formalin in PBS. Organ samples were trimmed and embedded in paraffin. Then, histological sections were prepared, stained with hematoxylin-eosin, and assessed under a light microscope.
